# Assessing Inter-Sensor Variability and Sensible Heat Flux Derivation Accuracy for a Large Aperture Scintillometer

**DOI:** 10.3390/s140202150

**Published:** 2014-01-27

**Authors:** Evan H. Rambikur, José L. Chávez

**Affiliations:** Department of Civil and Environmental Engineering Colorado State University, 1372 Campus Delivery, Fort Collins, CO 80523, USA; E-Mail: evan.rambikur@gmail.com

**Keywords:** large aperture scintillometer, sensor evaluation, eddy covariance, sensible heat flux

## Abstract

The accuracy in determining sensible heat flux (*H*) of three Kipp and Zonen large aperture scintillometers (LAS) was evaluated with reference to an eddy covariance (EC) system over relatively flat and uniform grassland near Timpas (CO, USA). Other tests have revealed inherent variability between Kipp and Zonen LAS units and bias to overestimate *H*. Average *H* fluxes were compared between LAS units and between LAS and EC. Despite good correlation, inter-LAS biases in *H* were found between 6% and 13% in terms of the linear regression slope. Physical misalignment was observed to result in increased scatter and bias between *H* solutions of a well-aligned and poorly-aligned LAS unit. Comparison of LAS and EC *H* showed little bias for one LAS unit, while the other two units overestimated EC *H* by more than 10%. A detector alignment issue may have caused the inter-LAS variability, supported by the observation in this study of differing power requirements between LAS units. It is possible that the LAS physical misalignment may have caused edge-of-beam signal noise as well as vulnerability to signal noise from wind-induced vibrations, both having an impact on the solution of *H*. In addition, there were some uncertainties in the solutions of *H* from the LAS and EC instruments, including lack of energy balance closure with the EC unit. However, the results obtained do not show clear evidence of inherent bias for the Kipp and Zonen LAS to overestimate *H* as found in other studies.

## 1. Introduction

It is known that estimation of evaporation and transpiration (evapotranspiration, ET) on varying spatial and temporal scales is important, whether on a field or farm scale for irrigation water management or a watershed scale for basin water management. Various technologies have been employed in this capacity on a research basis which include, but are not limited to, scintillometry, eddy covariance, and remote sensing. The eddy covariance (EC) method is a direct method of measuring latent (evaporative) and sensible heat fluxes using high frequency measurements of water vapor concentration, air temperature, and vertical wind speed. Despite the common issues with failing to close the surface energy balance [1,2], use of the EC method is prevalent likely because of the advantage of continuous, direct measurement of turbulent sensible (*H*) and latent (λ*E*) heat fluxes. It is also observed to be the common method for evaluation of Large Aperture Scintillometer-derived *H* and λ*E* [3–8]. Satellite- and airborne-based remote sensing methods are unique in their capability to provide land surface maps of information which can lead to production of ET raster maps. For validation of remote sensing ET, the Large Aperture Scintillometer (LAS) is considered an appropriate tool due to the relatively large spatial scale of measurement [8–10]. A LAS yields a spatial average of *H* (W·m^−2^) over path lengths up to 4.5 km, and relies on ancillary measurement of net radiation (*R_n_*, W·m^−2^) and ground or soil heat flux (*G*, W·m^−2^) to solve the land surface energy balance for λ*E* (W·m^−2^) as a residual.

The subject of this paper is restricted to the evaluation of the LAS estimation of *H* for a particular commercial model (Kipp and Zonen, Delft, The Netherlands) over a homogeneous surface. In particular, the precision of the Kipp and Zonen LAS was tested for three LAS units and accuracy was assessed with independent reference measurements from an EC system. A similar evaluation has been previously conducted by Kleissl and others [11]. The authors showed inter-LAS variability between 2% and 21%, expressed in terms of the *H* linear regression slope, for five Kipp and Zonen LAS units set up over a relatively flat grassland valley in New Mexico. In addition, comparison of *H* between LAS and EC was reported, but it was not readily clear which LAS was in overall better agreement with the EC reference. Peak *H* reported by the LAS units in the Kleissl study generally was between 250 and 300 W·m^−2^. Gowda [12] also showed preliminary results of a LAS inter-comparison study over a bare soil surface where three Kipp and Zonen LAS units showed 15%–20% deviation in *H*, in terms of the linear regression slope. Peak H for this study was roughly 500 W·m^−2^. In addition, Van Kesteren and Hartogensis [3] showed significant inter-LAS deviation in the air refractive index structure parameter (*C_n_*^2^) signal for four Kipp and Zonen LAS units set up over a grass field in the UK. From the same study the authors reported sensible heat flux bias somewhat larger than 30% in the Kipp and Zonen LAS relative to a Wageningen University (Wageningen, The Netherlands) designed LAS model, where peak *H* was generally less than 250 W·m^−2^. Kleissl and others [13] reported bias of 25% when comparing the Kipp and Zonen LAS to a Boundary Layer Scintillometer (BLS900, Scintec, Rottenburg, Germany) over an irrigated peanut field in Mexico, where peak H did not significantly exceed 100 W·m^−2^. Nonetheless, Brunsell and others [8] reported fair to good results for a Kipp and Zonen LAS compared with two EC systems over undulating grassland in Kansas. From these results, it is apparent that the performance of the Kipp and Zonen LAS is variable from instrument to instrument. Kleissl and others [11] discussed some possible reasons for the inter-sensor bias, and Van Kesteren and Hartogensis [3] also explained some internal deficiencies in the Kipp and Zonen LAS model. This study was undertaken to assess the precision and accuracy of the Kipp and Zonen LAS for estimation of sensible heat flux under the environmental conditions encountered in southeastern Colorado. The findings are compared and contrasted with the results of other similar studies mentioned above. Specifically, the results of the inter-LAS comparison of *H* and the comparison of LAS and EC *H* are presented.

## 2. Methods

### 2.1. Experimental Setup

**Figure 1.**
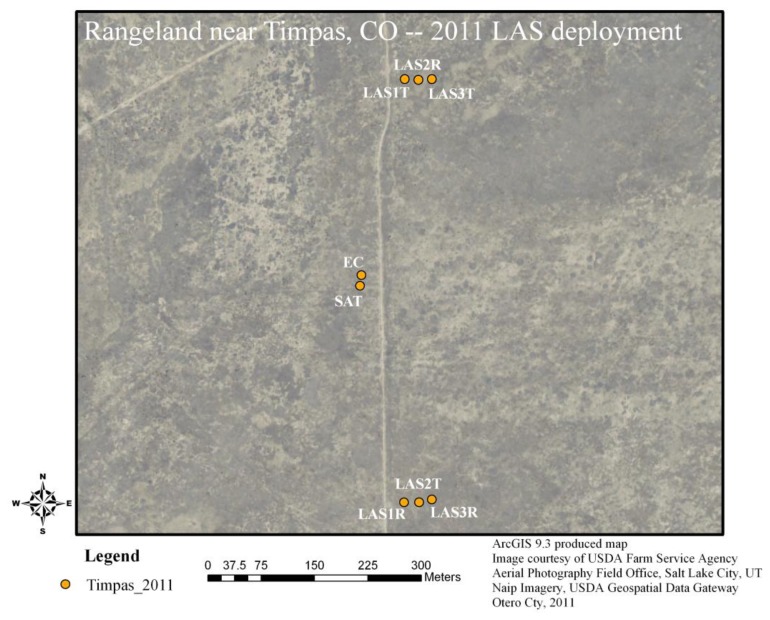
Aerial image overview of the Timpas grassland site. An access (dirt) road ran parallel to the LAS paths, in between the LAS units and the EC and SAT towers. LAS#T represents LAS transmitter and LAS#R represents LAS receiver.

**Figure 2.**
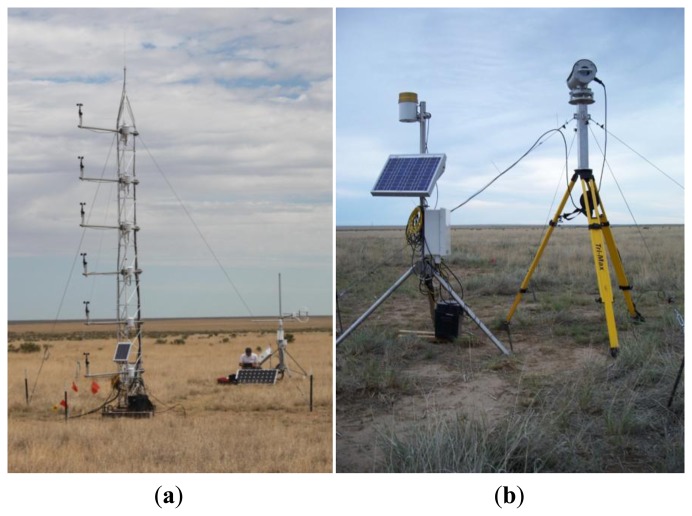
Photos of SAT and EC tower setup (**a**) and LAS-1 Receiver (LAS1R, Figure 1) setup (**b**). Left photo was taken on 21 July 2011 and right photo was taken on 3 August 2011.

During the summer of 2011, three Kipp and Zonen LAS units were installed at a relatively flat grassland site near Timpas, CO, USA (latitude 37.8173, longitude −)103.82304, elevation 1,350 m above sea level) and near the Comanche National Grasslands. The vegetation cover was mostly dry and did not seem to change significantly over the study period. There was a mix of short grass (approximately 9 cm) and tall grass (approximately 25 cm), along with occasional shrubs and cactus bushes (approximately 0.4–1.2 m;. The grass types in the historical climax plant community for the area were predominantly western wheatgrass, blue grama, and galleta grasses [14]. Approximately 51 mm of rainfall were recorded over the study period. An overview of the instrument deployment is shown in Figure 1. All LAS units were installed side by side at the Timpas site from 2 July 2011 to 3 August 2011. The transmitter and receiver units were mounted on top of a tripod with a custom extension, and anchored using four guy wires (Figure 2). The path length was approximately 600 m and LAS height as determined from LAS transmitter and receiver heights was approximately 2.25 m. There was approximately 20 m separation between each LAS transect, and in addition, the LAS-2 transect (transmitter to receiver) was inverted relative to that of LAS-1 and LAS-3, such that no risk of beam contamination was expected (Figure 1) (LAS beam width widens to approximately 1% of the path length upon reaching receiver [11]). Measurements from a surface aerodynamic profile (SAT) tower with six levels (each cross-arm about 1 m apart) were used to provide air temperature, relative humidity (Vaisala, Inc. HMP45C, Campbell Scientific Inc. (CSI), Logan, UT, USA), and wind speed (R.M. Young Wind Sentry 03101, CSI) as input for LAS data processing. In addition, an eddy covariance (EC, described later) system was installed adjacent to the SAT tower, both towers being approximately 40 m west of the closest LAS path at the approximate north-south path center (Figures 1 and 2). Both the SAT and EC systems were operational first on 8 July. At the SAT tower and at the LAS-1 receiver, ancillary instrumentation was installed to measure net radiation (net radiometer NR-Lite, Kipp and Zonen, CSI), radiometric surface temperature (infra-red thermometer IRT SI-111, Apogee, CSI), soil heat flux (soil heat flux plates, REBS HFT3, CSI,), shallow soil temperature (thermocouple T107, CSI), and shallow soil moisture (volumetric water content, CS616, CSI). Finally, barometric pressure (barometer CS106, Vaisala BAROCAP, CSI) and precipitation (rain gauge TE525, CSI) were measured at the LAS-1 receiver location.

### 2.2. LAS Theory and Processing Methods

The Kipp and Zonen LAS operates by propagating a near-infrared (880 nm) electromagnetic beam between a transmitter and receiver of equal aperture diameter. The signal is affected by “scintillations” or turbulence in the beam path caused (primarily) by variations in the air refractive index (*n*) [9]. The receiver captures the strength of the transmitted signal and correspondingly accounts for the variation of the signal strength in time. For turbulence in the inertial sub-range (applicable for a LAS), a unique relationship between signal variance (*σ*^2^*_lnI_*, where *I* is the signal) and the structure parameter of the air refractive index (*C_n_*^2^, m^−2/3^) exists as presented by Wang [15] (Equation (1)):

(1) $${C_{n}}^{2}=1.12{\sigma_{\text{lnI}}}^{2}D^{7/3}{L_{\text{LAS}}}^{-3}$$

where *D* is the LAS aperture diameter (0.152 m) and *L_LAS_* is the LAS path length (m). The *C_n_*^2^ parameter is affected by temperature, humidity, and (negligibly) barometric pressure fluctuations [9]; however for near-infrared wavelengths, temperature fluctuations are the primary contributor to *C_n_*^2^, such that the temperature structure parameter (*C_T_*^2^, K·m^−2/3^) can be approximately predicted from *C_n_*^2^ with additional input of only the Bowen Ratio (The Bowen Ratio is defined as the sensible heat flux (*H*) divided by the latent heat flux (λ*E*)) (β) [16,17] (Equation (2)):

(2) $${C_{T}}^{2}=\frac{T^{2}}{{A_{T}}^{2}}\cdot \frac{{C_{n}}^{2}}{{\left(1+\frac{0.03}{\beta }\right)}^{2}}$$

where $A_{T}=-0.78\cdot 10^{-6}\frac{BP}{T}+0.126\cdot 10^{-6}R_{v}q$ and *T* is air temperature (K), *BP* is barometric pressure (Pa), *R_v_* is the water vapor gas constant (461.5 J·kg^−1^·K^−1^), and *q* is specific humidity (kg·kg^−1^). For dry surfaces, the impact of β on *C_T_*^2^ is negligible. Subsequent application of Monin-Obukhov (M-O) similarity theory (MOST) permits the determination of the temperature scale (T_*_, K) from *C_T_*^2^ using an empirically derived similarity relationship (*f_T_*; Equation (3)) [9]:

(3) $$T_{*}={\left(\frac{{C_{T}}^{2}{\left(z_{LAS}-d\right)}^{\frac{2}{3}}}{f_{T}\left(\frac{z_{LAS}-d}{L_{mo}}\right)}\right)}^{0.5}$$

In Equation (3), *z_LAS_* is the effective LAS beam height (m), *d* is the zero displacement height (m), and *f_T_* represents the M-O similarity function for *C_T_*^2^ and T_*_. In order to finally determine *H* (Equation (6)), additional input of the friction velocity (u_*_, m·s^−1^) is required (Equation (4)). Both T_*_ and u_*_ are dependent (in a thermally stratified surface layer) on similarity functions of the buoyancy parameter (*z*/*L_mo_*), where *z* (m) represents the measurement height less the zero displacement height (*d*, m) and *L_mo_* is the Obukhov length (m) [18]. It is notable that *L_mo_* is also dependent on T_*_ and u_*_ (Equation (5)), thus requiring an iterative computation scheme to derive *H* from the LAS *C_n_*^2^ measurement:

(4) $$u_{*}=\frac{k_{v}\cdot (U_{2}-U_{1})}{ln\left(\frac{\left(z_{2}-d\right)}{\left(z_{1}-d\right)}\right)-\psi \left(\frac{z_{2}-d}{L_{mo}}\right)+\psi \left(\frac{z_{1}-d}{L_{mo}}\right)}$$

(5) $$L_{mo}=\frac{{u_{*}}^{2}T}{gk_{v}T_{*}}$$

(6) $$H=-\rho_{\text{air}}c_{p}u_{*}T_{*}$$

The equation for u_*_ above represents the logarithmic wind profile (LWP) model, where *k_v_* is the Von Karman constant (∼0.41), *U* represents horizontal wind speed (m·s^−1^) at two heights, *z*_1_ and *z*_2_ (m), and ψ represents the M-O similarity functions for u_*_. An alternative (more common) formulation of the LWP model using only one wind speed measurement is achieved by replacing *U*_1_ with zero (m·s^−1^) and *z*_1_-*d* with the momentum roughness length (*z_om_*, m) [18]. In Equations (5) and (6), *g* is the earth gravitational constant (9.81 m·s^−2^), *ρ_air_* is the moist air density (kg·m^−3^), and *c_p_* is the specific heat of dry air at constant pressure (∼1,005 J·kg^−1^·K^−1^). In this study, the formulations of *f_T_* (for *C_T_*^2^ and T_*_) from Andreas [19] and ψ (for u_*_) from Dyer [20] were used. These relationships (not shown here) have different formulation for unstable and stable atmospheric conditions; however LAS measurement does not account for atmospheric stability. Therefore, post-processing of raw data requires independent determination of the atmospheric stability condition. In this study, the sign of the air temperature profile from the SAT tower was used to determine the direction of sensible heat flux and thus the atmospheric stability conditions.

LAS and meteorological data were sampled at 1 Hz frequency and stored on a compact flash memory card using a Campbell Scientific (CR1000, CR3000) data logger. Downloaded 1 Hz data were subsequently averaged to 30 minute records. LAS *C_n_^2^* was computed from the mean and variance of the voltage (logarithmic) *C_n_*^2^ signal ( $U_{c_{n}^{2}}$, V). Friction velocity (u_*_) was computed, for the LAS, using the alternative one-level formulation (u_*__1-L_, Equation (4)). In addition, u_*_ was measured directly by the 3-D sonic anemometer at the EC tower and LAS data were also processed using this u_*_ solution (u_*__EC_; see Eddy Covariance Methods section). This resulted in two different solutions of *H* from each LAS unit. LAS path length (*L_LAS_*) was computed using handheld GPS (eTrex, Garmin, Olathe, KS, USA) readings which generally had an accuracy of ±4 m. The LAS effective beam height (*z_LAS_*) was taken as the average of the measured height of the transmitter and receiver units, neglecting elevation variability within the LAS path. Vegetation canopy height (*h_c_*, m) was sampled at different locations and different times during the test period. Momentum roughness length (*z_om_*, m) and zero displacement height (*d*, m) were determined from the estimated effective *h_c_* as 0.123 × *h_c_* and 0.67 × *h_c_*, respectively [21]. An initial Bowen Ratio (β) estimate was made for daytime and nighttime periods, where daytime period β varied between 0.5 and 1.5, depending on soil moisture conditions approximated from precipitation data. Net radiation (*R_n_*) and ground heat flux (*G*) time series for the site were determined using the average (For ground heat flux (*G*), the final value was taken from only one station since the values were found to be very similar for both stations) of two available stations for measurement; *G* was computed using the method described in the HFT3 SHF plate manual [22]. The *R_n_* and *G* data were used to compute β after the first determination of *H* from the LAS (*H_LAS_*) according to the energy balance method proposed by Green and Hayashi [7]. The time series energy balance β (β*_EB_*) was thus computed by Equation (7) after the first iteration of *H_LAS_*:

(7) $$\beta_{EB}=\frac{H_{LAS}}{R_{n}-G-H_{LAS}}$$

**Figure 3.**
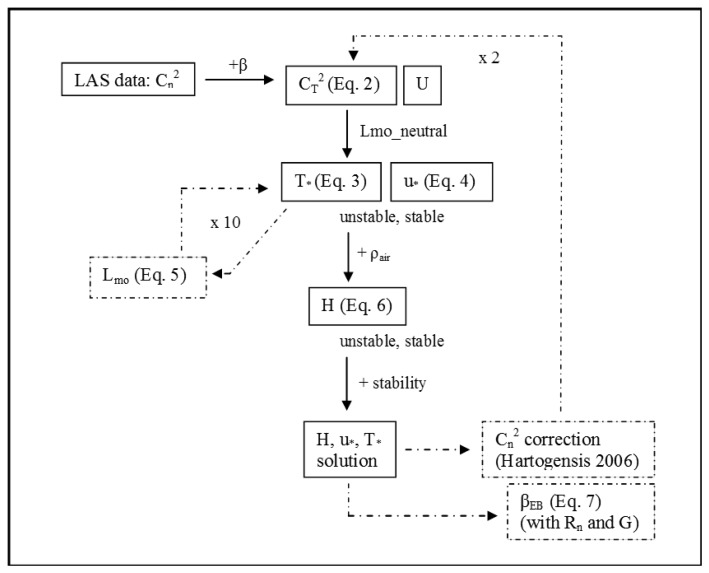
Simplified LAS data processing flowchart. Iterative processes are shown with a dashed line. “β” represents Bowen Ratio, “U” represents wind speed, “L_mo__neutral” represents the initial guess of *L_mo_* (± 1 × 10^5^ m), “ρ_air_” represents air density, and “β_EB_” represents the Bowen Ratio computed by energy balance.

In addition, a correction of the *C_n_*^2^ variable was performed based on Hartogensis [23] for contributions to the LAS signal from the dissipation range of turbulence. The correction of *C_n_*^2^ and the computation of β*_EB_* were performed iteratively with the computation of *H_LAS_* since the variables are codependent. The procedure was repeated until Δ*H_LAS_* between iterations converged to a near zero value (The final solution of *H_LAS_* was determined generally when the mean period of record Δ*H* was less than 1 W·m^−2^ and the count of Δ*H* values larger than 5 W·m^−2^ was small), which was typically achieved by the third iteration of *H*. In Figure 3 a flowchart of the LAS processing methods is shown.

### 2.3. Eddy Covariance Methods

Surface layer fluxes can be determined using the covariance of the vertical wind speed with a variable of interest (e.g., temperature) provided that appropriate conditions exist, including horizontal surface homogeneity, flux stationarity, and presence of turbulence [24]. This is the basis for the EC method, which requires high frequency (at least 10 Hz) sampling of all variables measured. In this study, a 3-D sonic anemometer (CSAT3, CSI) was used to measure the wind speed in three orthogonal directions (*u*, *v*, *w*; m·s^−1^) and the sonic temperature (T_s_, K). An ultraviolet krypton hygrometer (KH20, CSI) was used to measure the water vapor concentration (or specific humidity, q, kg·kg^−1^). Measurements were made at 10 Hz frequency and processed for averaging intervals of 30 min using EdiRe^®^ software [25]. Processed output data included friction velocity (u_*_) and sensible (*H*) and latent (λ*E*) heat flux. Data were controlled and corrected in the following order within the processing software operation: signal de-spiking, coordinate rotation [26], KH20 signal lag, KH20 oxygen correction [27], frequency response correction [28], density correction [29], sonic temperature correction [30], and steady state and integral turbulence tests [31]. Observation of the EC flux output revealed a tendency for underestimation of the available energy by approximately 20% during the daytime. When comparing *H* between the LAS units and the EC, no adjustment was made to the EC *H* solution for the lack of energy balance closure. This issue is further addressed in the discussion section.

### 2.4. Data Quality Control and Filtering

During processing LAS data were controlled using two QC checks; data were filtered for low signal (Demodulated signal less than 50 mV; this filter was not applied to a subset of LAS-2 data, after the unit had become almost completely misaligned, in order to test the effect on LAS performance for such a case) and signal saturation [32]. Periods with precipitation were filtered out. These initial filters were applied to the inter-LAS and the LAS to EC comparison data. Subsequent filters were applied only for the LAS to EC comparison: Periods with temperature gradient less than 0.2 °C between two arms on the SAT tower were filtered out from the LAS dataset to avoid risk of wrong determination of the atmospheric stability. Periods with u_*_ less than 0.15 m·s^−1^ were also filtered out (LAS and EC) to avoid conditions with poorly developed turbulence. In addition, stable period LAS data with low wind speed demonstrated problems with non-convergence of *L_mo_*, resulting in solutions of u_*_, Lmo, and H drawing near to zero; these periods were excluded from comparison. EC data were filtered if the wind came from a 60° sector directly behind the tower to avoid disturbance caused by the tower structure. The results of the steady state and integral turbulence (IT) tests proposed by Foken and Wichura [31] were used to filtered EC data as follows: if horizontal wind speed (*u*) and temperature (*T*) data violated steady state requirements by more than 30%, data were excluded; if *u* and *T* data violated IT requirements by 50% (not 30%), data were excluded (The relaxed IT filter was implemented to allow more data for comparison, since the IT filter using a 30% violation limit would have been very restrictive).

## 3. Results

### 3.1. Inter-LAS Comparison

The LAS units at the study site were set up within a period of a few hours and, following manufacturer recommendations, the units were aligned (transmitter to receiver and receiver to transmitter) and the power was set at the transmitter to achieve a signal strength at the receiver of 50% (−)375 mV). Interestingly, the analog power requirement to achieve 50% signal strength was 50 for LAS-1, 72 for LAS-3, and 110 for LAS-2. Within the same day of setup, the alignment of LAS-3 had already dropped just below 40%. After one week of operation, the signal strength of both LAS-2 and LAS-3 fell to approximately 25%, deteriorating further to less than 20% over the subsequent two weeks. During a site visit on 21 July (2011), the alignment was restored in LAS-2 and LAS-3, although a storm the same afternoon appears to have caused the subsequent (complete) misalignment observed with LAS-2. Units LAS-1 and LAS-3 remained aligned during the remainder of the data collection period. Table 1 below summarizes the approximate (range of) signal strength for each LAS during the periods defined by the alignment issues discussed above.

**Figure 4.**
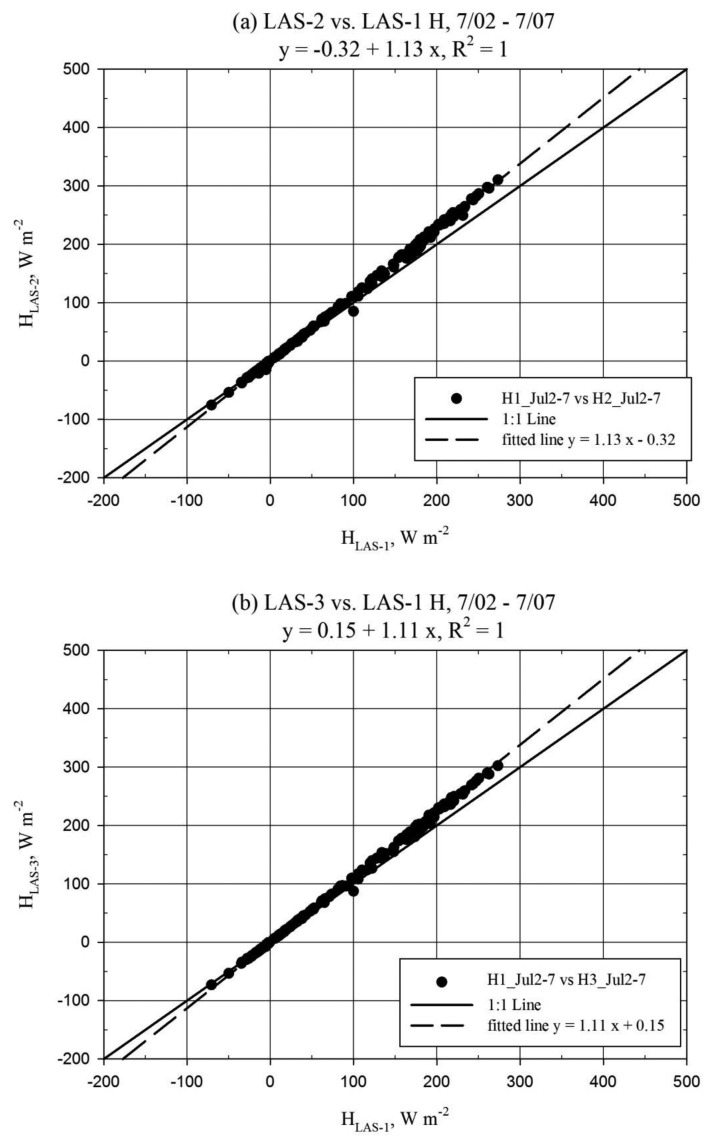

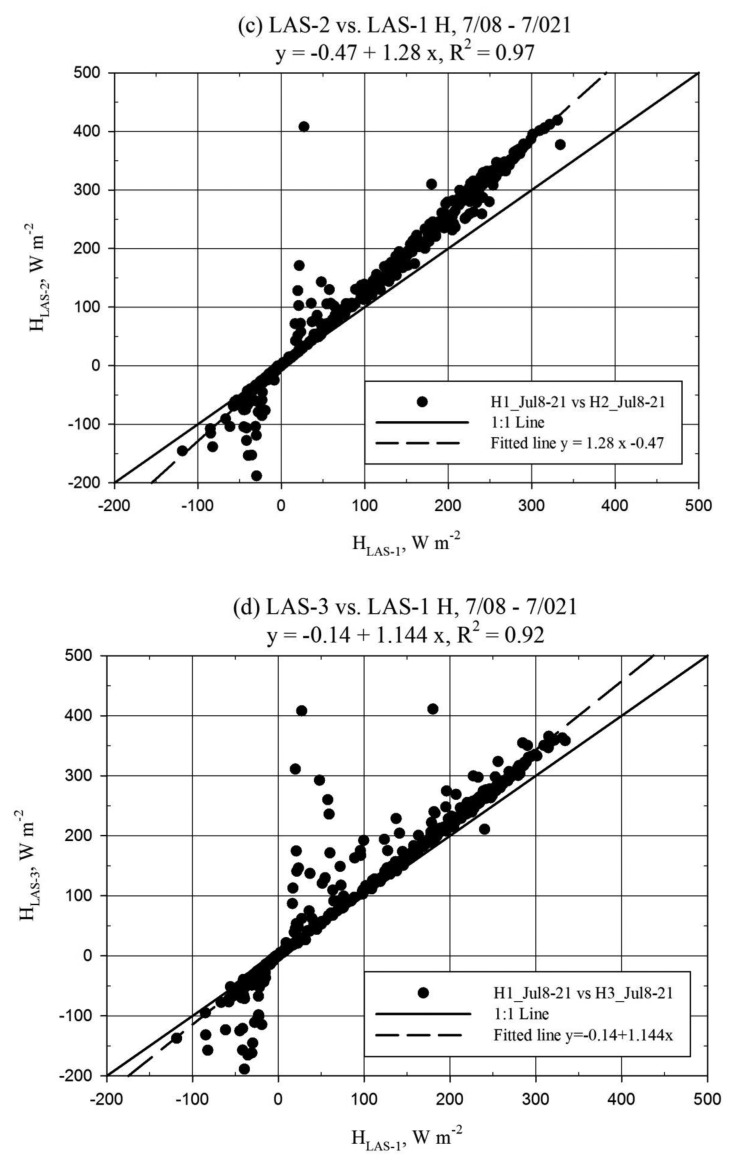

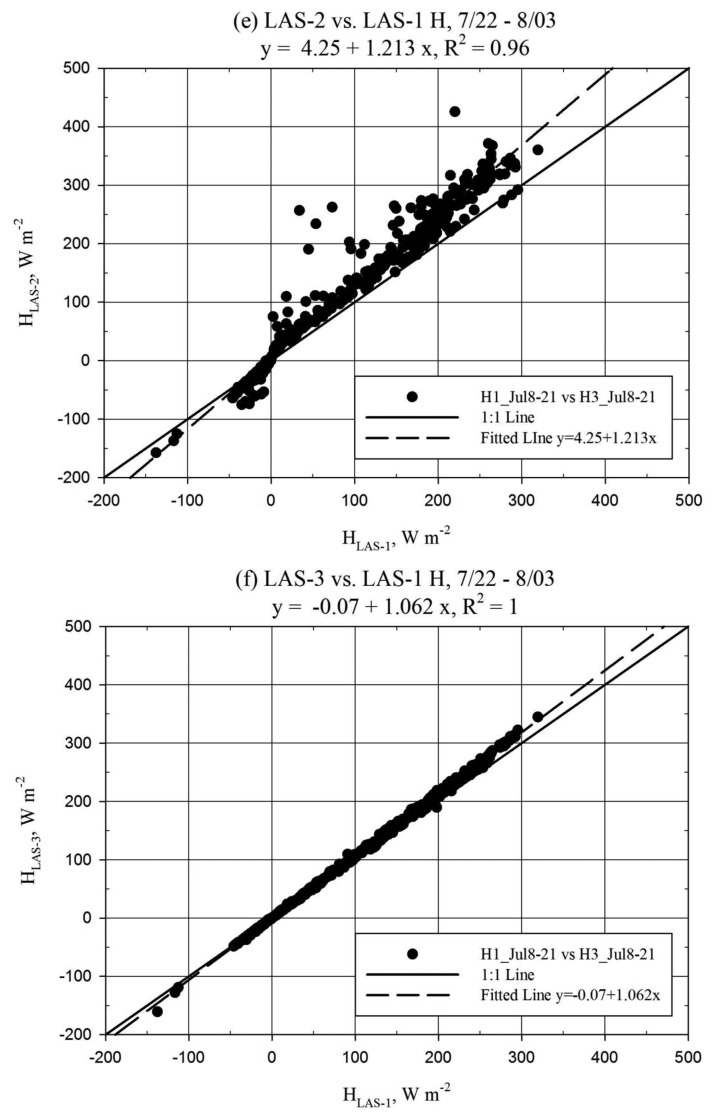
Regression plots for sensible heat flux (*H*, W·m^−2^) of LAS-1, LAS-2, and LAS-3, for 2–7 July (**a**,**b**), 8–21 July (**c**,**d**), and 22 July–3 August (**e**,**f**); LAS-1 is reference. Results filtered for precipitation only. Dashed line represents best-fit linear regression; solid line represents the 1:1 relationship. Regression slopes were statistically significantly different from 1.0 in each case (Table A1).

**Figure 5.**
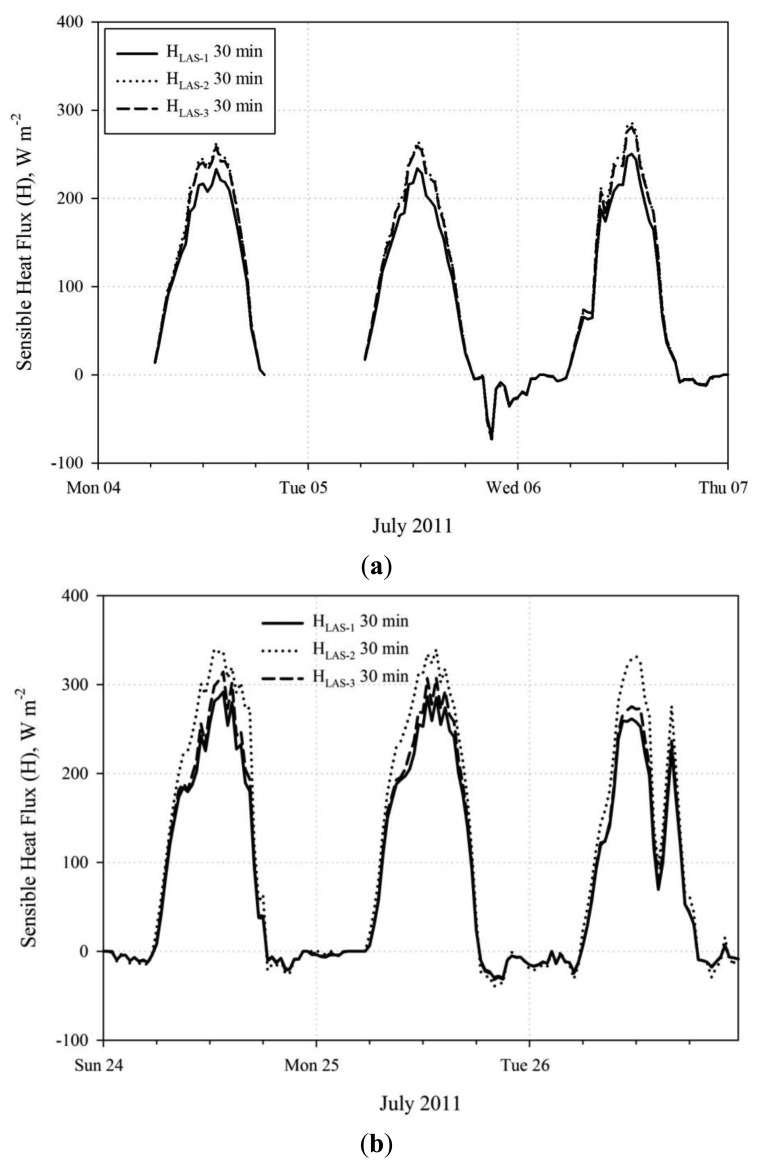
Time series plots of sensible heat flux (*H*, W·m^−2^) for LAS-1, LAS-2, and LAS-3 for data subsets from 4–6 July, representing good LAS alignment (**a**) and 24–26 July, representing poor alignment in LAS-2 and good alignment in LAS-1 and LAS-3 (**b**). Figure 5a represents data shown in Figure 4a,b. Figure 5b represents data shown in Figure 4e,f.

The results for the inter-LAS *H* comparison are presented for three data subsets, as shown in Table 1, based on the above discussion of the alignment issues over the period of record. Figure 4 shows the relationship between the *H* solutions from each LAS unit using scatter plots and Figure 5 shows the *H* solution time series for sample days. Comparison of *H* was also facilitated using summary statistics including the mean bias error divided by mean of the absolute value of observation *H* (MBE/|Ō|). This parameter is an alternative relative deviation parameter which can be used if mean relative deviation is considered unrealistic due to effects of unbounded relative deviation for near zero values of (e.g.,) *H*. Reference (|Ō|) data were taken from LAS-1 for the inter-LAS comparison, since LAS-1 did not become misaligned during the study period. MBE and |Ō| represent period averages in W·m^−2^. The inter-comparison results represent LAS data processed using the u_*__1-L_ method. Additional dataset statistics beyond those reported here are provided in a table in the Appendix, referenced as Table A1 in the report body. For the first week when all units were well aligned, there was very little scatter between the *H* solutions for any of the LAS units (Figure 4a,b). However, there was a mean bias (MBE/|Ō|) of approximately +11% for LAS-2 relative to LAS-1 and +9% for LAS-3 relative to LAS-1. Units LAS-2 and LAS-3 were very well correlated with little bias. Following the decrease in signal strength of LAS-2 and LAS-3 observed on 8 July, scatter increased between all LAS units and bias increased between LAS-2 and LAS-1 (Figure 4c,d). The MBE/|Ō| between LAS-2 and LAS-1 increased to 24% and, in addition, disagreement in *H* pattern between LAS units was apparent for afternoon/nighttime periods associated with larger wind speeds, generally about 8 m s^−1^. Further, an MBE/|Ō| of 7% was observed between LAS-2 and LAS-3, making apparent that the slip in alignment did not affect LAS-2 and LAS-3 the same. Notably, the trend between LAS-3 and LAS-1 did not appear to change after the 8 July slip in alignment despite the observed increase in scatter (Figure 4d). After the complete misalignment of LAS-2 late 21 July along with the improved alignment of LAS-3, the level of scatter and bias between LAS-2 and LAS-1 remained similar to the prior subset, but the scatter and bias between LAS-3 and LAS-1 were reduced (Figure 4e,f). The MBE/|Ō| value between LAS-3 and LAS-1 was reduced from 18% to 6%, which was lower even than the 9% bias observed during the first subset. It is notable that despite the signal strength of LAS-2 being near zero, the general diurnal pattern in *H_LAS_*_-2_ was similar to that of LAS-1 and LAS-3 (Figure 5b). Furthermore, the deviation between LAS-2 and LAS-1 *H* was not larger than for the prior period when LAS-2 had approximately 20% signal strength. For each comparison period, mean *H* values between all LAS units were significantly different (Table A1).

### 3.2. LAS to EC Comparison

**Figure 6.**
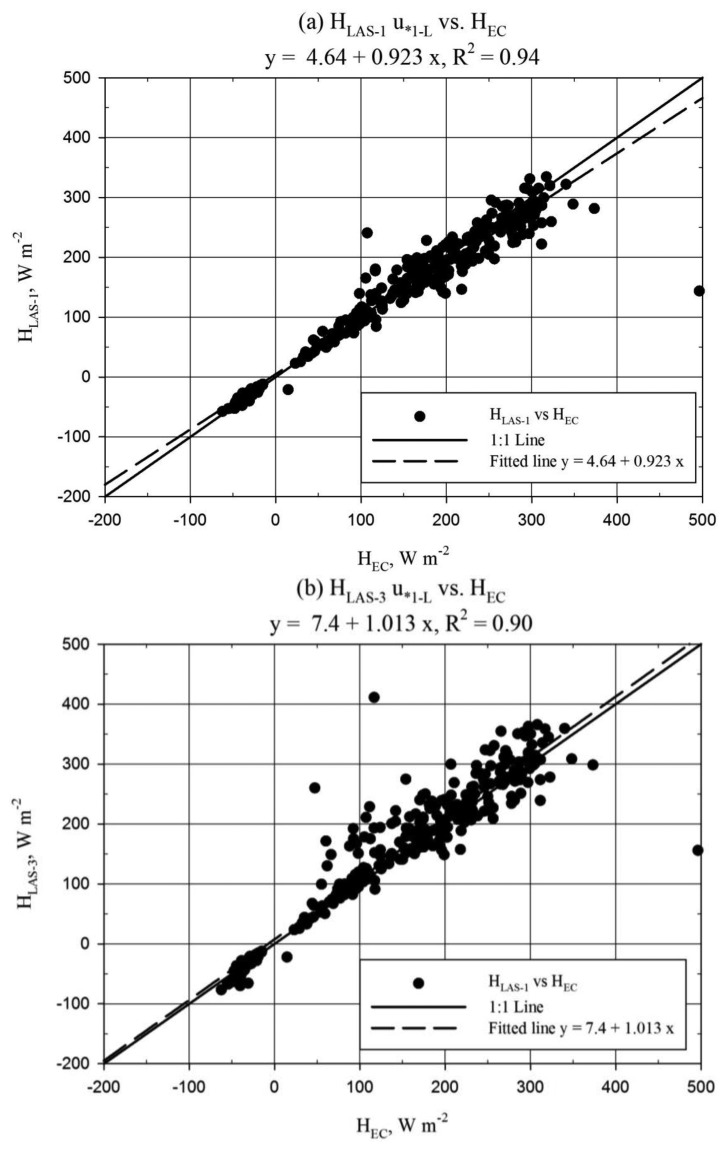
Regression plots for sensible heat flux (*H*, W·m^−2^) comparing LAS-1 *versus H_EC_* (**a**) and LAS-3 *versus H_EC_* (**b**). Dashed line represents best-fit linear regression; Solid line represents the 1:1 relationship; Results from u_*__1-L_ method.

The LAS to EC comparison was performed from 9 July to 2 August, based on the availability of EC data, and statistics were not divided based on the above “alignment” periods. The LAS-2 and EC comparison was not included, since LAS-2 was significantly impacted by the 8 July slip in alignment (Figure 4c) and the unit was completely out of alignment after 21 July. Recall that only LAS-1 maintained consistent (good) alignment over the period of record. The scatter plots of LAS and EC *H* for the u_*__1-L_ method are shown in Figure 6 below.

**Figure 7.**
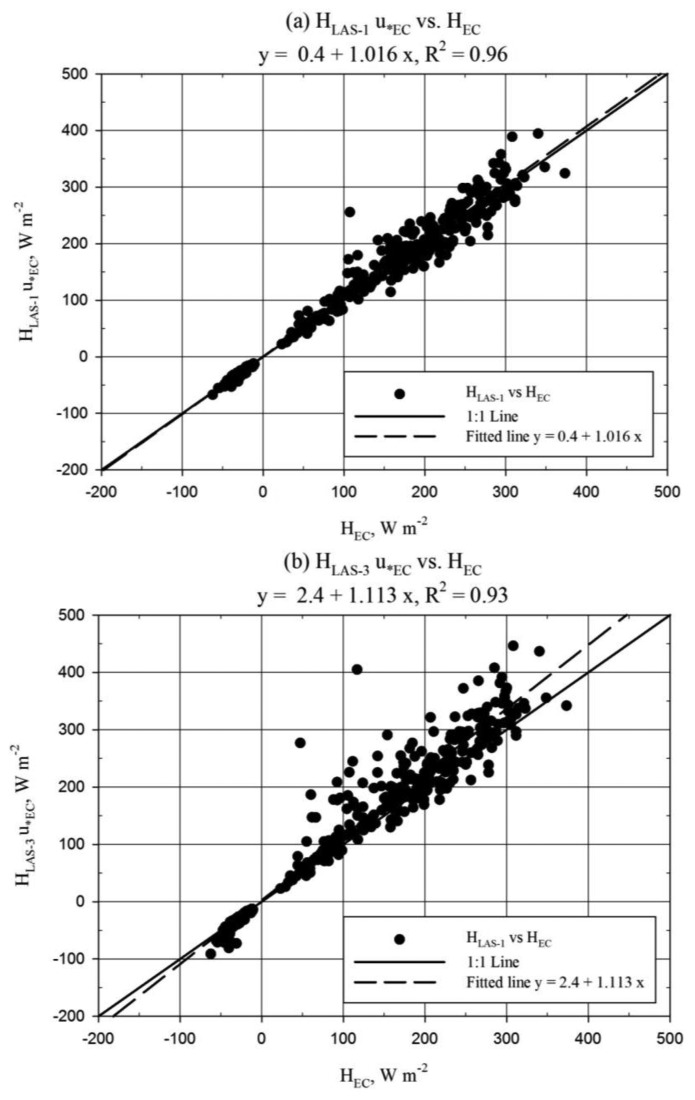
Same as Figure 6, for u_*__EC_ method.

Good correlation, better than 90%, was observed between *H_LAS_* and *H_EC_* (Figure 6). More significant scatter between *H_LAS_*_-3_ and *H_EC_* could be explained by the misalignment during the 8–21 July period. *H_LAS-_*_1_ was observed to underestimate *H_EC_* by approximately 4% (MBE/|Ō|) while *H_LAS-_*_3_ exhibited bias to overestimate *H_EC_* by roughly 8% (MBE/|Ō|). The scatter plots therefore must be interpreted carefully, since Figure 6b suggests *H_LAS-_*_3_ to have little bias toward *H_EC_*. In fact the regression slope of 1.01 (Figure 6b) was not significantly different from 1.0 (Table A1). Although not shown here, the u_*__1-L_ solution was observed to trend slightly lower than the EC u_*_ solution (u_*__EC_) (regression slope = 0.93, *R^2^* = 0.85). Comparison of *H_EC_* and *H_LAS_* processed using u_*__EC_ is shown below in Figure 7. This comparison provides a better evaluation of the LAS sensor since u_*_ is an external variable in the LAS solution. The regression slope comparing EC and LAS-1 (Figure 7a) was not significantly different from 1.0 (Table A1). Furthermore, mean *H* was not significantly different between EC and LAS-1 processed using u_*__EC_ (Table A1).

It is apparent from Figures 6 and 7 that *H_LAS_* with u_*__EC_ tended to be larger than *H_LAS_* with u_*__1-L_, to be expected considering the relationship between u_*__1-L_ and u_*__EC_. For the u_*__EC_ case, *H_LAS-_*_1_ shows little bias with respect to *H_EC_*, while *H_LAS-_*_3_ overestimates *H_EC_* (Figure 7). This observation is confirmed by deviation statistics, which show *H* overestimation biases of 3% and 15% for LAS-1 and LAS-3, respectively, with respect to the EC (MBE/|Ō|).

## 4. Discussion and Conclusions

### 4.1. Inter-LAS Comparison, Good Alignment

LAS units are well aligned when the transmitter is focused on the receiver aperture and *vice versa*. For periods of good LAS alignment the results from this study are considered comparable to the studies of Kleissl and others [11], Gowda [12] and Van Kesteren and Hartogensis [3], since there was no mention of misalignment in these references. As has been shown in the above references, we also found inter-LAS deviation in *H*. Specifically, regression slope biases of 6% (LAS-3) and 13% (LAS-2) were observed, which fall in the range found by Kleissl and others [11]. Van Kesteren and Hartogensis [3] suggested the deviations observed by Kleissl and others [11] could be attributed mainly to (internal) detector alignment issues. The lens focal point detector in the Kipp and Zonen LAS transmitter and receiver units might be particularly prone to erroneous alignment, where even if the LAS is physically well-aligned, the unit will be in poor alignment. In their study, they showed that four Kipp and Zonen LAS units overestimated *C_n_*^2^ with respect to a research grade LAS, with regression slopes varying between 1.35 and 3.40. Recall that *C_n_*^2^ is the primary output of the LAS and variability in *C_n_*^2^ will correspond to variability in *H*. Based on the observations from these reference studies, it appears that a Kipp and Zonen LAS may be internally misaligned whether or not this is manifested in abnormal power requirements for the LAS. However, in this study it was observed that the inter-LAS deviation in *C_n_^2^* followed the pattern of power requirements among the LAS units. *H_LAS-_*_2_ was found to be greater than *H_LAS-_*_3_ which was greater than *H_LAS-_*_1_, and power requirements for LAS-2 were greater than LAS-3 which were greater than those for LAS-1 (LAS-3 (physical) alignment was slightly better for part of the 22 July–3 August period than for the 2–8 July period, such that the LAS-3 to LAS-1 bias is considered only sensor-induced from 22 July to 3 August, while the LAS-2 to LAS-1 bias is considered only sensor-induced from 2–8 July, *i.e.*, *H_LAS-_*_2_ = 1.13·*H_LAS-_*_1_ and *H_LAS-_*_3_ = 1.06·*H_LAS-_*_1_ (Figure 4)). It is suspected that if LAS-2 and LAS-3 were returned to the manufacturer for maintenance, the detector alignment would be found in error, and that correction of this issue would result in a relative reduction in *C_n_*^2^ (and *H*). This can be supported by the results of Kleissl and others [11], who showed dramatic reduction in *H* from one LAS after repair of the detector alignment. They also observed this LAS had a significantly higher power requirement than the other units. One further issue is an apparent calibration drift observed in LAS-1. A manufacturer-recommended calibration check of the receiver unit electronics was conducted and showed good calibration for LAS-2 and LAS-3, but underestimation of the reference signal for LAS-1. The manufacturer was not contacted concerning the potential impact of the calibration drift in LAS-1 on *C_n_*^2^. However, the impact on *C_n_*^2^ may have been small considering the good comparison between LAS-1 and the EC unit (Figure 7). Nonetheless, it is recommended (and good practice) to periodically have the LAS sensor recalibrated by the manufacturer to avoid potential impacts caused by any calibration drift. From the results here, it is concluded that the Kipp and Zonen LAS is prone to inter-sensor deviation in the estimation of *H*, as has been found by Kleissl and others [11] and Van Kesteren and Hartogensis [3]. This outcome may be explained perhaps especially by detector alignment issues. In addition to periodic LAS recalibration it is thus recommended to have the detector alignment of the sensor verified with the manufacturer. However, even with the detector alignment confirmed, it would be preferable to compare the Kipp and Zonen LAS to an independent and trusted reference before solo deployment in the field.

### 4.2. Inter-LAS Comparison, Poor Alignment

Further discussion is warranted based on the increase in bias and scatter noted after misalignment occurred in LAS-2 and LAS-3. The regression slope for *H_LAS-_*_2_ to *H_LAS-_*_1_ increased from 1.13 to 1.28 from the 2–7 July to the 8–21 July period. For *H_LAS-_*_3_, the trend with respect to LAS-1 did not apparently change for the same period, which may have been because LAS-3 alignment was (already) not perfect during the 2–8 July period. However, the bias and scatter between LAS-1 and LAS-3 *H* were reduced after the 21 July realignment of LAS-3. The manufacturer warns that if LAS alignment is not good, the receiver may “see” the edge of the transmitted beam, negatively impacting the *C_n_*^2^ signal. Thus, it is suspected that this “edge” effect is the cause for the increased bias resulting from the LAS misalignment. In addition, periods were noted where there was pattern disagreement between the *H_LAS_* solutions corresponding with the 8–21 July misalignment period (see scatter in Figure 4c,d). These disagreements tended also to correspond with higher wind speeds in the afternoon and evening, generally when horizontal wind speeds were about 8 m·s^−1^. Consequently, the scatter is attributed to increased noise resulting from vibration of the LAS units caused by higher wind speeds. This presumes that the wind speed affected more the misaligned units since they may have been more loosely secured than the well-aligned LAS-1. The manufacturer suggests setting a lower limit for the demodulated signal of (e.g.,) 50 mV in order to ensure good quality of the *C_n_^2^* signal (The demodulated signal scale is actually negative, so a limit of 50 mV would require the signal to be less (more negative) than −50 mV). The signal strength for LAS-2 and LAS-3 during the 8–21 July period did not drop below 100 mV, which suggests that a fixed lower limit value is insufficient to avoid errors in *H* caused by LAS misalignment. Further, after complete misalignment (signal strength near zero), LAS-2 performed similarly compared to the previous period when alignment was better (Figure 4c,e). Therefore, it is concluded that good alignment of the LAS transmitter and receiver is a prerequisite to ensure good data quality. In addition, it is recommended to restore alignment in the LAS units as soon as possible after an observed drop in signal strength. It is further recommended to provide a stable base for the sensor and fix the alignment securely to avoid noise cause by vibrations.

### 4.3. LAS Performance, EC Reference

The performance of the LAS with respect to the EC was found to be good for LAS-1, with relative bias of only 3% for *H_LAS_* processed using u_*__EC_ (MBE/|Ō|). Recall that *H_LAS-_*_3_ was biased larger than *H_LAS-_*_1_ between 6% and 14% in terms of regression slope over the period of comparison to the EC, which result was reflected in *H_LAS-_*_3_ overestimating *H_EC_* (Figure 7). Further, on the basis of the 13% regression slope bias observed for *H_LAS-_*_2_ greater than *H_LAS-_*_1_ from 2–8 July, the performance of LAS-2 with respect to the EC can be inferred, that *H_LAS-_*_2_ would have likely exhibited overestimation bias with respect to *H_EC_* somewhat larger than that of *H_LAS-_*_3_. These results support our conclusion that detector misalignment affected the performance of LAS-2 and LAS-3. However, some other factors warrant mention. The effective LAS beam height was not rigorously determined and it is suspected that the estimated height may have been slightly underestimated. This would have led to an underestimated solution of *H_LAS_*. Further, we observed the LAS-1 electronics to underestimate the reference signal in a calibration check. This underestimation was roughly 48 mV in the voltage *C_n_*^2^ signal for path length settings close to that used at Timpas. This may have resulted in underestimation of *H* by LAS-1. One further issue is the accuracy of the *H_EC_* solution, which is important because the performance of *H_LAS_* is evaluated with *H_EC_* as a standard. The observation of typically 20% lack of energy balance closure shared between *H_EC_* and λ*E_EC_* suggests the possibility that *H_EC_* was underestimated. Some common assertions regarding the lack of energy balance closure include the following, (a) that the source areas of the energy balance components do not match and/or the energy balance neglects components which may be significant such as heat storage and/or advection and (b) that the eddy covariance method suffers from “missing” low/high frequency eddy structures due to the temporal and spatial scale of the turbulent eddies [1,2]. The accuracy of the measured *R_n_* and *G* for the site could partially explain the apparent lack of energy balance closure, however even with perfect values of *R_n_* and *G*, the lack of energy balance closure displayed by the EC system would not likely have been solved. Since the site was relatively flat and uniform in terms of vegetation type and moisture, there was no expectation for horizontal advection of energy as an additional flux input. Further, the relatively low vegetation density and height suggests safe neglect of canopy energy storage. There was, however, observation of slight positive correlation between energy balance closure and wind speed (or u_*_), suggesting that the energy balance closure was better for more turbulent conditions. Recently, Kochendorfer [33] suggested that the vertical wind speed tends to be underestimated by non-orthogonal sonic anemometers, including the CSAT3. If this was the case at Timpas, the u_*_, *H*, and λ*E* solutions from the EC would have been underestimated. All the factors discussed here suggest *H* may have been greater than observed with the LAS and EC instruments. The uncertainty introduced with the unknowns in this study is realized. Nonetheless, the principal conclusions from this study related to the inter-comparison of LAS *H* are presumed to remain valid, being in part independent of the comparison between LAS and EC *H*.

## Figures and Tables

**Table 1.** Demodulated signal strength given in analog (0%–100%) format for the three LAS units during the inter-comparison study. 8 July and 21 July divide the study period due to the changes in alignment for one or more of the LAS units observed on those days.

**Unit ID**	**2–8 July**	**8–21 July**	**22 July–3 August**
LAS-1	48%	50%	50%
LAS-2	48%	16%–25%	0%
LAS-3	37%	16%–25%	37%–45%

## Appendix

**Table A1.** Descriptive and deviation statistics for LAS inter-comparison and LAS-EC comparison *H* values (W m^−2^). *t*_R1 represents *t* statistic to test for regression slope different from 1.0; *t*_means represents *t* statistic to test for difference between period mean *H* values. *t*_crit. represents critical *t* value for alpha equal to 1%. *p* values represent probability of *t* values being less in magnitude than *t*_crit.

**Descriptive Statistics**									

**Parameter**	**2 July–3 August 2011**			**9 July–3 August 2011, *u*_*_***_1-L_*			**9 July–3 August 2011, *u*_*_***_EC_*		
	**LAS-1**	**LAS-2**	**LAS-3**	**EC**	**LAS-1**	**LAS-3**	**EC**	**LAS-1**	**LAS-3**
***n***	1373	1373	1373	380	380	380	388	388	388
**Mean**	71.1	88.9	78.4	114.6	110.4	123.5	109.0	111.2	123.8
**Standard Error**	2.8	3.5	3.1	6.3	6.0	6.7	6.2	6.4	7.1
**Median**	21.0	32.9	24.6	123.3	127.9	151.3	112.3	120.2	144.6
**Standard Deviation**	102.8	129.0	116.2	123.4	117.5	131.4	121.3	125.8	140.4
**Minimum**	−137.6	−225.7	−269.8	−62.4	−116.5	−127.9	−62.4	−120.9	−133.0
**Maximum**	334.3	425.5	410.8	496.4	334.3	410.8	373.3	394.2	446.2
**Skewness** ^1^	**0.65**	**0.60**	**0.54**	0.09	−0.03	−0.04	0.10	0.10	0.08
**Kurtosis ^1^**	**−0.98**	**−0.89**	**−0.83**	**−1.22**	**−1.41**	**−1.36**	**−1.39**	**−1.34**	**−1.30**

**Deviation Statistics, LAS Inter-comparison**									

**Parameter**	**2–7 July 2011, *n* = 203**			**8–21 July 2011, *n* = 617**			**22 July–3 August 2011, *n* = 553**		
	**1–2**	**1–3**	**2–3**	**1–2**	**1–3**	**2–3**	**1–2**	**1–3**	**2–3**
**slope**	1.13	1.11	0.98	1.28	1.14	0.90	1.21	1.06	0.84
***y*-int.**	−0.32	0.15	0.49	−0.47	−0.14	−0.57	4.25	−0.07	−0.87
***R*^2^**	0.999	0.999	1.000	0.971	0.923	0.971	0.964	0.999	0.963
***t*_R1**	46.38	42.41	−15.14	32.05	10.79	−16.00	21.23	46.19	−22.24
***t*_crit.-R1**	2.60	2.60	2.60	2.58	2.58	2.58	2.58	2.58	2.58
***p*_R1**	3 × 10^−109^	3 × 10^−102^	5 × 10^−35^	3 × 10^−133^	6 × 10^−25^	2 × 10^−48^	1 × 10^−73^	1 × 10^−191^	1 × 10^−78^
***t*_means**	−11.50	−11.98	6.63	−11.91	−6.07	8.76	−14.43	−14.81	12.66
***t*_crit.-means**	2.60	2.60	2.60	2.58	2.58	2.58	2.58	2.58	2.58
***p*_means**	7.2 × 10^−24^	2.5 × 10^−25^	2.9 × 10^−10^	1.4 × 10^−29^	2.2 × 10^−9^	1.9 × 10^−17^	2.8 × 10^−40^	4.8 × 10^−42^	2.0 × 10^−32^

**Deviation Statistics, LAS-EC Comparison**									

**Parameter**	***u*_*_***_1-L_*, ***n* = 380**		**u_*__EC_, *n* = 388**						
	**EC-LAS1**	**EC-LAS3**	**EC-LAS1**	**EC-LAS3**					
**slope**	0.92	1.01	1.02	1.11					
***y*-int.**	4.64	7.40	0.40	2.40					
***R*^2^**	0.938	0.904	0.961	0.926					
***t*_R1**	−6.35	0.74	1.55	7.08					
***t*_crit.-R1**	2.59	2.59	2.59	2.59					
***p*_R1**	6 × 10^−10^	0.458	0.121	7 × 10^−12^					
***t*_means**	2.68	−4.24	−1.71	−7.17					
***t*_crit.-means**	2.59	2.59	2.59	2.59					
***p*_means**	0.008	3 × 10^−5^	0.088	4 × 10^−12^					

1. Bold red text represents potentially significant skewness and kurtosis as described in [34], suggesting departure from normal distribution.
